# Hepatectomy and pneumectomy combined with targeted therapy for primary hepatic neuroendocrine carcinoma: Case report and review of the literature

**DOI:** 10.3389/fsurg.2022.920276

**Published:** 2022-07-15

**Authors:** Keyu Huang, Zhujing Lan, Weitao Chen, Jianyong Zhang, Jilong Wang, Hai Zhu, Banghao Xu, Ling Zhang, Tingting Lu, Ya Guo, Zhang Wen

**Affiliations:** ^1^ Department of Hepatobiliary Surgery, The First Affiliated Hospital of Guangxi Medical University, Nanning, China; ^2^ Department of Radiology, The First Affiliated Hospital of Guangxi Medical University, Nanning, China; ^3^ Department of Ultrasound, The First Affiliated Hospital of Guangxi Medical University, Nanning, China

**Keywords:** liver, neuroendocrine carcinoma, targeted therapy, gene sequencing, case report

## Abstract

Primary hepatic neuroendocrine carcinoma (PHNEC) manifests as a rare type of liver tumor. PHNEC is not specifically clinical or radiographical and is often misdiagnosed and mistreated. Here, we present a case report of PHNEC in a 50-year-old woman who was admitted to our department with concealed pain in the right upper abdomen. The initial diagnosis was a probable hepatic space-occupying lesion with tumor bleeding. The patient was subjected to a partial right hemihepatectomy, cholecystectomy, partial resection of the lower lobe of the right lung, partial resection of the diaphragm, and resection of the right perirenal fat sac to alleviate her symptoms. After surgery, gene sequencing was performed to determine the possible cause of the condition. However, five months after discharge, the patient was hospitalized again because of retroperitoneal and peritoneal multiple metastases. Nine months after surgery, the patient died. This case is likely to aid in furthering our understanding of PHNEC to improve the future diagnosis and treatment of this disease.

## Introduction

Neuroendocrine tumor (NET) refers to a tumor that originates from neuroendocrine cells (1) and is also known as a “carcinoid.” The incidence rate of this condition is extremely low when compared with other cancers, and it can occur in any part of the body. The highest incidence is in the digestive system and the lungs. Seeing the primary disease in the liver is rare, and its occurrence in this organ probably means it was transferred there. Primary hepatic neuroendocrine carcinoma (PHNEC) accounts for only 0.3% (2) of all NETs. The incidence rate of primary liver malignancies is 0.46% (3), and most of them are not functional. Distinguishing between metastatic and primary liver cancer *via* ultrasound and other imaging methods is difficult. Most patients are treated for tumor growth and complications. In this case, the patient was diagnosed with a hepatic space-occupying lesion at admission and was later diagnosed with PHNEC *via* pathology after an operation.

## Case presentation

A 50-year-old woman was admitted to the hospital on March 22, 2021, because of dull pain in the right upper abdomen for more than 1 month, and the pain was concentrated in the region of the liver for more than 10 days. Before admission, computed tomography (CT) scans acquired in another hospital revealed the presence of multiple space-occupying lesions in the right lobe of the liver. A physical examination was performed after admission. The abdomen was flat and soft with no tenderness or rebound tenderness over the entire region. There was a palpable area situated 8 cm below the liver costal, which was soft with no percussion pain in the liver area. Palpations were not observed under the spleen costal, her bowel movements were normal, bowel sounds normal, and there was no edema in both her lower extremities. Routine hospital tests were performed: C-reactive protein 35.37 mg/L. Liver function tests: Child A grade; ICG 15R = 1.7%, HBsAg (−). Carcinoembryonic antigen and alpha-fetoprotein were normal. Past surgical history: The patient had undergone a total isthmusectomy and a bilateral subtotal thyroidectomy in April 2019.

**Figure 1:**
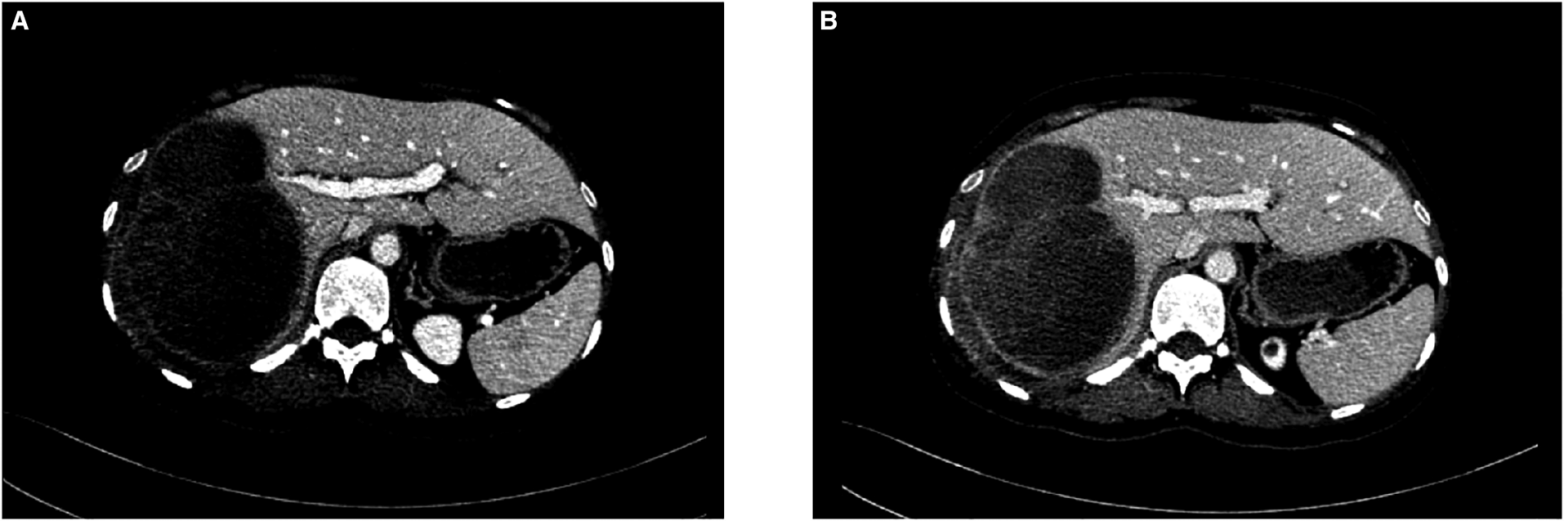
Preoperative abdominal CT findings (**A**) arterial phase (**B**) venous phase.

**Figure 2:**
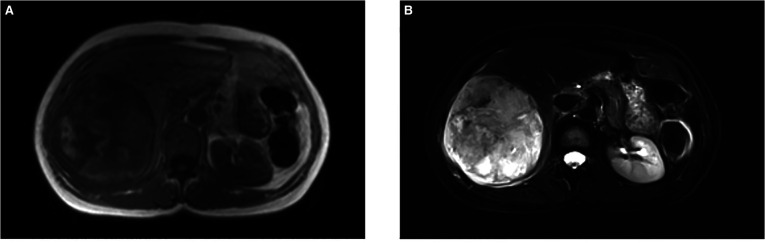
Findings of magnetic resonance imaging (MRI). (**A**) T1-weighted image of the nodule showed an iso-intensity lession. (**B**) T2-weighted image of the tumor presented a slightly higher intensity. (F) Diffusion-weighted image of the tumor was a marked hyper–intensity.

**Figure 3:**
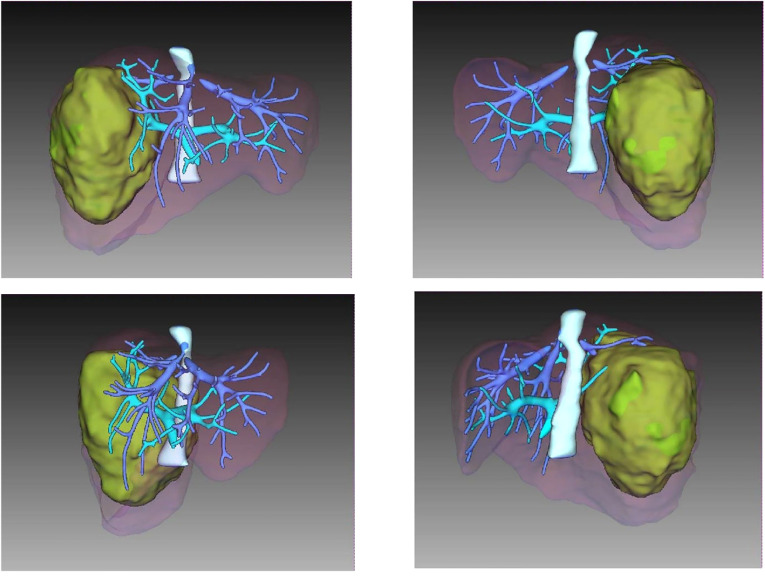
Three dimensional imaging of tumor and vessel added with IQQA.

A CT scan showed a cystic-solid mass (16.2 × 10 × 10.7 cm) on the right lobe of the liver, but no abnormalities were found in the intrahepatic and extrahepatic bile ducts and gall bladder. Furthermore, there was a malignant tumor on the right lobe of the liver, with ruptured hemorrhage and hepatic adenoma (Figure 1). MRI + MRCP showed large and nodular signals on the right lobe of the liver, with evident boundaries. The bile duct in the right lobe of the liver was compressed and displaced, and the intrahepatic bile duct was positioned to the left. However, the lumen of the right hepatic ducts and the common bile duct were not dilated. A tumor on the right lobe of the liver was observed to be ruptured and bleeding (Figure 2). The three-dimensional images of the tumor and vessels with the addition of intelligent qualitative and quantitative analysis based on the CT results are shown in Figure 3.

### Operative procedures

**Figure 4:**
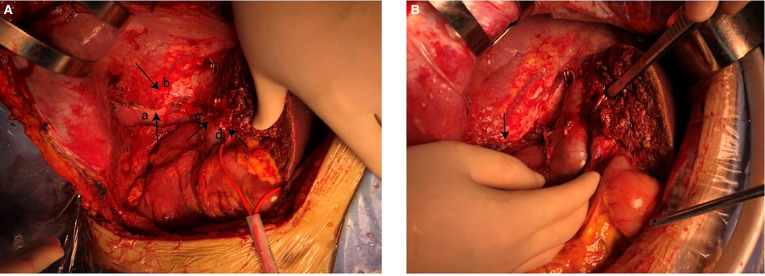
(**A**) resected partial diaphragm and lung tissue. (**a**) The right lung exposed after removal of part of the diaphragm. (**b**) Diaphragm. (**c**) Remnant of right hepatic vein. (**d**) Remnant of middle hepatic vein. (**B**) Diaphragm after repair.

**Figure 5:**
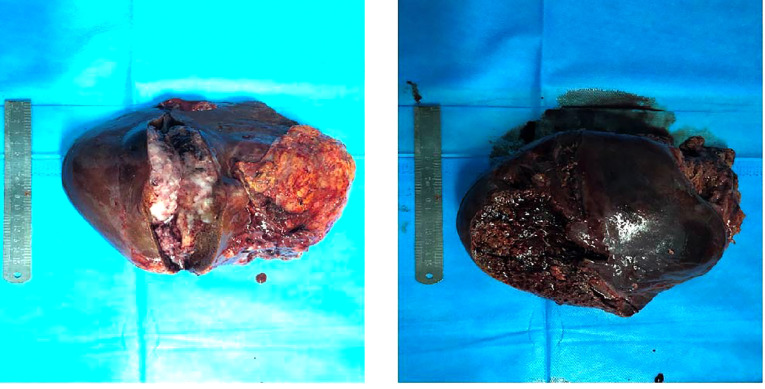
Resectted tumor specimens.

After general anesthesia, a reverse L-shaped incision was used. The abdominal cavity was explored, the gallbladder was removed, and intraoperative ultrasound and intraoperative fluorescence imaging devices were used to detect the number and location of the tumor. The tumor was located in the right lobe of the liver. The capsule of tumor was incomplete, the boundary was unclear, there was ischemic necrosis and bleeding in the center of tumor. The tumor invaded the diaphragm, the size was about 15 cm × 15 cm. An anterior approach hepatectomy was used, a vascular occlusion device was placed at the first porta hepatis, and the liver parenchyma was transected by harmonic ace and bipolar coagulation. The Glissonean pedicle was clipped and cut off, combined with the right caudate lobe resection, the right deltoid ligament, and the right coronary ligament was separated, right semihepatectomy was completed. Because the tumor invaded the diaphragm, and the diaphragm was partially excised 10 cm × 10 cm (Figure 4). Part of the right lower lung tissue was resected. The diaphragm wound was sutured to form a repair and closed thoracic drainage was placed at the same time. The anterior fat sac of the right kidney was resected togetherly. Intraoperative resection specimens are shown in Figure 5.

**Figure 6:**
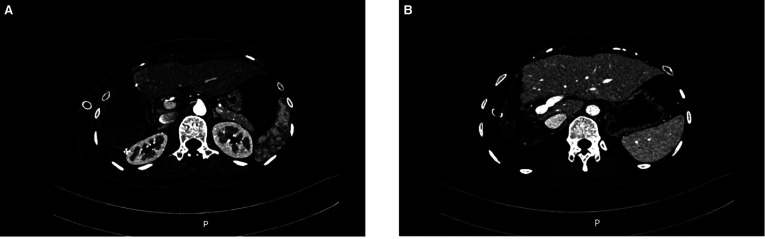
Postoperative CT findings. (**A**) Arterial phase (**B**) Venous phase.

The operation time was 217 min, and the blood loss was 300 ml. No autologous blood was transfused during the operation. The postoperative CT scan confirmed that the tumor had been resected (Figure 6). The patient's condition gradually improved. The patient was fed on the 2nd postoperative day and was discharged from the hospital on the 8th day. After discharge, the patient was treated with sunitinib for targeted drug therapy.

### Postoperative pathology report

**Figure 7:**
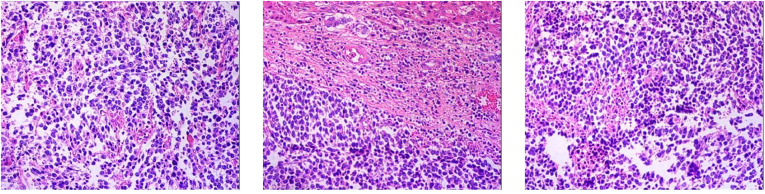
Pathological result of postoperative HE staining.

1. Neuroendocrine carcinoma of the right lobe of the liver (small cell carcinoma) 2. Right renal capsule fibro-fatty tissue infiltration/metastatic neuroendocrine carcinoma 3. Invasion of diaphragmatic striated surface connective tissue/metastatic neuroendocrine carcinoma (Figure 7). Immunohistochemical staining assessment: the right lobe of the liver was positive for CD56, CgA, and Syn and weakly positive for CD99 and Ki-67 (80%). The expressions of CK, EMA, Bcl-2, CD34, and SMA were all negative. The evidence suggested the presence of neuroendocrine cancer. The right renal capsule was positive for Syn and Ki-67 (50%), which supported the diagnosis of invasive/metastatic neuroendocrine carcinoma.

Gene sequencing was performed with a sample of the patient's tissue after the surgery. Totally, 14 gene variants were detected, among which 2 variants with potential clinical significance were in the CCND1 and TP53 genes.

The patient returned to the hospital for re-examination a month after discharge. A CT scan showed the presence of multiple masses occupying the posterior and upper parts of the pancreatic head and the right abdominal and retroabdominal cavities, with a maximum size of 3.1 × 2.7 × 5.2 cm, which was indicative of metastases. A 5-month follow-up ultrasound after discharge showed a solid mass in the right renal first hepatic hilum areas and in the upper segment of the abdominal aorta, which was considered evidence of a malignant tumor. The mass had increased in size in comparison with the previous scan. The patient was readmitted to the hospital because of the presence of an abdominal mass. A CT examination showed that there were multiple metastases in the retroperitoneum and abdominal cavity, the largest being 9.0 × 8.5 cm, and a portal vein tumor thrombus had formed. After mass resection of the right hepatic lobe, the patient was found to have multiple metastases in the peritoneum and retroabdominal cavity. After admission, symptomatic and supportive treatments were given. After completing the relevant examinations, the patient was advised to obtain comprehensive treatment in the Medical Oncology Department, where she was evaluated for surgery and other possible regimens. The patient and her family were informed about the condition and the related risks; they expressed their understanding of the risks but asked to be discharged from the hospital. After 9 months of follow-up, the patient died.

## Discussion

NET is a type of rare tumor that originates from the neuroendocrine cells. According to the World Health Organization classification of digestive system tumors in 2019 (5th edition), NETs can be distinguished into well-differentiated and poorly differentiated tumors (NECs) based on genetic evidence and differences in clinical, epidemiological, and histological features and prognosis. In addition, NET can be divided into G1, G2, and G3, which refer to low-, moderate-, and high-grade malignancy, respectively, based on its proliferative activity (4). NETs can occur in any part of the body, but they are more common in the digestive system, including the stomach, intestines, and pancreas. Most of the NETs in the liver are derived from metastatic lesions, and there are relatively few primary hepatic NETs, accounting for only 0.3% of all such tumors (2). Edmondson was the first to report a primary hepatic NET in 1958 (5). In the past decade, just over 200 cases of this disease have been reported at home and abroad. Most cases involve a single tumor, and the pathogenesis tends to be unclear. Some scholars believe that they originate from neuroendocrine cells of the bile duct epithelium in the liver (6) or from stem cells dedifferentiated from other malignant hepatocytes and transformed into neuroendocrine cells (7). These alterations may result from gene deletions and mutations.

Previous case reports on PHNEC show that it is more common in middle-aged people and women (8–10). PHNEC usually does not have specific clinical symptoms, and abdominal pain is a common symptom in many cases (11). An analysis of patients with PHNEC showed that 73.3% showed clinical symptoms, of which 65% had abdominal pain (12). Approximately 5% of the patients also had manifestations of carcinoid syndrome, such as skin redness, shortness of breath, and diarrhea (13). The lesions of primary hepatic NETs are often isolated and are commonly seen on the right lobe of the liver, with extended vascular invasion and less distant metastases. In this case, the tumor had invaded the right renal capsule and the diaphragm. Moreover, there were peritoneal and retroperitoneal metastases after discharge, which were very rarely noted in previous reports.

PHNEC is difficult to diagnose clinically. The main reason for this difficulty is that the clinical manifestations in the patients are nonspecific. The results from imaging techniques, such as ultrasound, CT, and MRI, can easily be confused with other types of liver tumors, although they are indicative of this disease. The imaging studies usually reveal hypoechoic, hyperechoic, or mixed-echo lesions, with rings around the tumors. Color echoes can be misdiagnosed as hemangioma based on the blood flow echo signals (14). In some reports, a plain CT scan could show a primary liver NET with low mass density and marginal enhancement, and the center of the mass would not be enhanced because of necrosis (15). In an MRI scan, T1W shows up as a low signal and T2WI as a high one. After enhancement, the arterial phase would demonstrate obvious nodular or marginal enhancement. The portal vein and delayed phases would show obvious filling continuous enhancement, and there could be an enhancement capsule in the latter. This finding tends to be different from the “fast in and fast out” enhancement normally seen in hepatocellular carcinoma or the “progressive” mild-to-moderate enhancement seen in cholangiocarcinoma. Therefore, NETs can be distinguished from other types of liver tumors by their location, shape, and portal vein enhancement.

Common serological tumor markers, such as AFP, CEA, and CA 19–9, have shown no diagnostic significance for patients with PHNEC. However, when 17 patients with PHNEC were analyzed, it was found that CA 19–9 and ferritin possessed a certain diagnostic value for the disease (16). The diagnosis of PHNEC mainly depends on tissue biopsy and subsequent immunohistochemistry. PHNEC can only be considered after eliminating the possibility of NET metastasis in other organs of the digestive tract. Immunohistochemistry is an essential method for the diagnosis of NETs, including positivity for chromogranin A (CGA), synaptophysin (SYN), and CD56. CGA is a characteristic marker for the diagnosis of PHNEC, and it exhibits the highest specificity. This marker is used to identify neuroendocrine cells and the source of tumors. These endocrine indexes reveal the characteristics of nerve cell differentiation. Ki-67 is a nuclear antigen related to proliferation and is of immense significance for the gradation of the tumor. Ki-67 serves as a practical reference index to evaluate the degree of malignancy and can guide diagnosis and prognosis. The higher the Ki-67 index, the lower the survival rate and the worse the prognosis of the patients. In the relevant literature, the positivity rates for SYN, CGA, and CD56 of PHNEC are >80% (1).

Surgery is currently the primary treatment for PHNEC (12). Knox et al. (10) and Iwao et al. (11) conducted studies on the survival of patients with PHNEC and showed that the 5-year survival rate of patients undergoing surgical therapy was >50%. However, owing to the rarity of the disease, its clinical features and treatment outcomes are poorly understood when compared with other types of tumors, which results in a poor prognosis. In this case, the tumor was found to have invaded the right renal capsule and diaphragm; hence, parts of the diaphragm and the fat sac in front of the right kidney were resected. When tumors metastasize to distant sites, the treatment becomes more complicated for patients with PHNEC because of unclear pathological mechanisms. Therefore, it is necessary to further explore the pathogenesis in these patients and accumulate experience in the diagnosis and treatment of the disease. Transcatheter arterial chemoembolization (TACE) could potentially be used in patients with unresectable disease or distant metastases (17, 18). The results obtained so far show that the median survival time of patients with PHNEC after TACE surgery is 39.6 months and that the 5-year survival rate is 35.5%, which significantly prolongs the survival time of the patients. Presently, there is no relevant clinical research report on targeted therapy for this disease; therefore, there is no consensus on what kind of treatment plan should be adopted. Currently, most physicians follow the guidelines for the treatment of gastrointestinal NET.

The concept of individualized tumor therapy has gradually emerged with advancements in medical science. In the past, individualized tumor therapy was narrowly referred to as targeted therapy. This concept has now been extended to the combination of multidisciplinary comprehensive treatment cooperation groups based on various factors, such as tumor stage and differentiation, performance status, and clinical course of the patients (15). Patients can now be offered a more targeted and comprehensive treatment plan.

The use of genetic sequencing was explored for this patient, and the genetic report suggested two meaningful mutations in the CCND1 and TP53 genes. CCND1 encodes a protein that transmits extracellular growth signals to the cell cycle by activating cyclin-dependent kinase (CDK)4 and CDK6, also called cyclin D1. The amplification and overexpression of CCND1 in human tumors lead to carcinogenicity. The CCND1 gene has been amplified and overexpressed in breast cancer tissue. The gene interacts with estrogen and hormone receptors in various forms to promote tumor growth. Its presence suggests that a patient is at a high risk for breast cancer. Studies on the relationship between CCND1 and genetic susceptibility to digestive system tumors are still in progress. TP53 encodes p53 protein, which is a tumor suppressor as well as a transcription factor that stimulates the cells to respond to carcinogens, such as DNA repair and apoptosis. TP53 is the most common mutant gene in human cancers, and its absence is closely related to the development of most cancers. Mutations in TP53 are mostly missense mutations that occur in the conserved regions of exons 5, 6, 7, and 8, and these are often seen in various hematological tumors and skin cancers. At present, gene mutations and the pathogenesis of NETs are being studied.

According to the genetic variations seen in patients, the gene sequencing suggests that the targeted drug that may benefit patients is piperacillin and the recommended chemotherapeutic drugs are platinum and vinblastine. According to the NMPA/FDA guidelines, based on the type of cancer (e.g., NET) that the patient currently suffers from, certain drugs such as sunitinib and everolimus are recommended. After discharge, the patient has been taking sunitinib for targeted drug therapy.

According to the consensus guidelines for diagnosing NETs proposed by the North American Neuroendocrine Tumor Society, platinum-based drugs (including cisplatin and carboplatin) and etoposide, have shown good results. Hence, they are listed as standard drugs for PHNEC (19). It has been reported that a patient began to use carboplatin and etoposide adjuvant chemotherapy 1 month after the operation. However, the patient died of systemic recurrence, which included residual liver metastasis 15 months after the operation (15). Therefore, chemotherapy can be used as an initial treatment option for PHNEC (19). Studies have shown that for patients with advanced PHNEC, platinum-based chemotherapy (etoposide combined with cisplatin/carboplatin, EP/EC) is more effective than TACE (20). In general, it is essential to evaluate tumor proliferation based on immunohistochemical results and the necessity for surgical resection (21).

In this case, the patient's clinical manifestations were not particularly indicative and there was no diagnosis of hepatitis or liver cirrhosis. Additionally, the tumor markers were normal, and the imaging results suggested the presence of solid liver space-occupying lesions. Therefore the possibility of hepatic NETs was considered. The diagnosis of patients with PHNEC needs to be supported more effectively with a tissue biopsy and immunohistochemistry. Surgical treatment would be the first choice, but a more comprehensive treatment is also a good choice for those without surgical indications or complex treatment methodology. The increased use of advanced gene detection technology should be encouraged in all patients to widen our knowledge of PHNEC. Therefore, in the future, a combination of gene sequencing and imaging and immunohistochemical techniques can aid in achieving a better diagnosis in patients with PHNEC. Such a diagnosis would, in turn, refine the therapeutic options available to the physician for managing the patients with this condition.

## Data Availability

The raw data supporting the conclusions of this article will be made available by the authors, without undue reservation.
